# 

**Published:** 1820-04-01

**Authors:** 


Armstrong, Dr. John, Southampton-row
Ashwell, S. Esq. Westminster lying-in Hospital
Bartlett, P. Esq. Nottingham
Bisset, C. E. Surgeon, Peckham
Bowen, Dr. J. Carmarthen
Braine, J. W. Esq. Oxford
Bradley, O. Esq. Surgeon, Royal Westmoreland Militia, Appleby
Broughton, S. Esq. Falmouth
Buchanan, Mr. Surgeon, Terrace, City Road
Byass, Mr. Surgeon, Crookfield
Callow, W. C. 20th light dragoons
Camm, W. Esq. Nottingham
Chambers, Mr. Charles, Member of the Royal College of Surgeons, London, Leamington Spa
Chilvers, Samuel, Esq. New Burlington-street
Child, Mr. Assistant Surgeon, Madras Establishment
Child, Mr. T. S. Bengal Medical Estab.
Clarke, T. Esq. Newman's-row, Lincoln's Inn Fields
Cooke, Dr. John, Fellow of the Royal College of Physicians, Gower-street
Cooper, Astley, Esq. Spring Gardens
Crawford, Dr. Bath
Creesy, Mr. Surgeon, Eden Bridge, Surrey
Darrack, Mr. Philadelphia
Davidson, Dr. J. M. Hanley, Staffordshire
Davies, Mr. W. S. G. Surgeon, Poland street
Davies, Mr. Surgeon, Conduit-street
Dickenson, Mr. Milcham
Dickenson, Mr. Sloane-street
Fearn, Mr. Thomas, Huntsvile, State of Alabama, United States
Foot, Mr. Jessé, Jamaica
Foot, Mr. George Forrester, Jamaica
Forbes, Dr. Chatham
Fosbrooke, Mr. London
Franklin, H. Esq. Surgeon of the 37th Regiment, Montreal
Freshfield, Mr. Philip William, M. R. C. Surgeons, London, Oakley, Essex
Gilbert, Mr. James, Surgeon, Brompton, Rochester
Gordon, Dr. Blenheim
Gordon and Graham, London
Gorman, Mr. Surgeon, Castle-street, Leicester-square
Grainger, Mr. St. Savior's Church-yard, London
Grey, Sir Thomas, M. D. London and Ramsgate
Green, Mr. William, Surgeon, Durham
Green, Dr. George, Yarm, Yorkshire
Green, Mr. Josephus Septimus, Surgeon Durham
Guy, Mr. John, Member of the Royal College of Surgeons, London
Hall, Mr. Robert, Durham
Howard, Dr. William, Baltimore, United States
Johnston, Mr. Somerset-st. Portman-sq.
Johnson, James, M. D. Bengal Medical Establishment
Kennedy, Mr. Charles, (St. Thomas's Hospital) Alverstone, Lancashire
Litchfield, Mr. Twickenham
Leggett, Mr. William, St. Thomas's Hospital
Lynn Regis Medical Book Society
Mathews, Mr. Thomas Leman, H. C. S. Prince Regent
Medical Book Society, Kingston, Jamaica
Millington, Mr. Titchfield-street
M'Knight, Dr. W. G. Jamaica
M'Leod, Dr. Ophthalmic Hospital
M'Millen, Mr. Surgeon, R. N.
Moffatt, Mr. Panton-square
Moffat, Mr. Surgeon, Long-lane
Money, Mr. Hanover-street, Hanover-square
Morris, Mr.
Morrison, Mr. W. Surgeon, H.P. 12th Foot, Bristol
Philip, Dr. A. P. W. Worcester.
Rankin, Mr. Marchmont-street
Riley, Edward, Esq. Sydney, New South Wales
Rodgers, Mr. James, Settle, Yorkshire
Royal Institution, Albemarle-street
Rutherford, Dr.
Stanton, Dr. R. N. Moulton, Northamptonshire
Steart, Augustus W. Bath
Stevenson, Mr. Member of the Royal College of Surgeons, London
Squib, Mr. Saville-row
Taylor, Dr. David, Wotton-under-Edge, Gloucestershire
Tickell, Mr. E. Burton, Somerset
Thornton, Dr. Edward, Stroud
Todd, J. T. Esq. Havre de Grace
Turner, Mr. Surgeon, New-street, Baker-street
Uwins, David, M. D. in exchange for Medical Repository
Walker, Dr. Sanderson, St. Michaels, Azores
Walton, Mr.
Walton, George, Esq. Surgeon, East Ind. Comp. Service
Wardrop, James, Esq. F.R.S.E. Charles-street, St. James's-square
Warwick, Mr. R. N. Newfoundland.
Whiteing, the Reverend Mr. G.B. Hitching, Herts
Whimper, Mr. G. M. Surgeon, Tillingham, Essex

*In the Press.*

Dr. JAMES JOHNSON has in the Press, Third Edition, entirely re-written, and greatly improved,

### A TREATISE ON THE LIVER,

### INTERNAL ORGANS, and NERVOUS SYSTEM;

### Pathological and Therapeutical.

☞ This Work will exhibit a complete Delineation of the Organic, as well as Functional Diseases of the Liver, the Heart, and other internal Organs, being entirely recomposed, and greatly enlarged. It will appear in July next.

### INDEX.

A

ACCRETIONS, tuberculated 169
Adams, Sir W. on cataract 86
Address to the profession 321
Ætius on animal food in diabetes 366
Albers, Dr. case of aneurism 47
Ancle, dislocations of 557
Aneurism, Dr. Alber's case of 47
——— Dr. Port's case of 49
——— Mr. Travers on 249
——— of the hepatic artery 434
——— of the heart 489
Angina pectoris, case of 262
Apoplexia cephalica, Dr. Stoker on 244
Apoplexy, Dr. Watts on 437
——— Dr. Cheyne on 439
———, pulmonary 485
———, Dr. Cook on 505
Apparatus for the cure of epilepsy 218
Archer, Dr. on paracentesis thoracis 250
Armstrong, Dr. on typhus 401
Arnott, Mr. on stricture 273
Arteria innominata tied 147
Arteriotomy in hydrophthalmia 142
Arteries, Dr. Parry on 201
———, Mr. C. Bell on 210
Auscultation, Laennec on 461

B.

Ballingal, Dr. on dysentery 31
——— on fever 27
——— on liver complaints 41
Bampfield, Mr. on dysentery 303
Baron, Dr. on tuberculated accretions 169
Barry, Dr. on intestinal worms 266
Bateman, Dr. on diseases of London 333
Bell, Mr. Charles, on the circulation 201
——— on the teeth 604
Berri Berri, Mr. Ridley on 435
Belcombe, Dr. on phlegmatia dolens 497
Bile not the cause of cholera 552
Black, Dr. on diseases of the brain 231
———, Dr. clinical reports 357
Blair, Dr. his death and dissection 133
Blane, Sir G. on medical logic 186
Bleeding, local, efficacy of 114
Blood, on its circulation 201
———, Mr. Thackrah on 296
———, Mr. Wilson's lectures on 432
Blood-letting, Dr. Welsh on 278
Bologna, state of medicine in 589
Bones, Mr. Howship on 48
Bostock, Dr. on ophthalmia 601
Brain, Dr. Black on diseases of the 231
Brande, Professor, on Gravel 291
Brigham, Mr. on pyloric ulceration 445
Bronchiæ, dilatation of 470
Bronchitis in a child, fatal 136

C.

Cæsarian operation, successful 273
Canada, medical topography of 293
Carbutt, Dr. cases of morbid anatomy 136
Carmichael, Mr. extirpation of the parotid gland 253
Carpue, Mr. on lythotomy 393
Cartilaginous fistulæ of the lungs 467
Cataract, Sir William Adams on 86
Cautery, actual, M. Maunoir on 248
Cephalea 597
Chapman, Mr. expulsion of a blighted fœtus 259
Chest, diseases of 461
Cheyne, Dr. on apoplexy 439
——— on hydrocephalus 505
Children, diseases of 151
Cholera epidemic of India 547
Chorea 644
Christie, Dr. on Berri Berri 436
Circulation, Parry and Bell on the 201
Clarke, Sir Arthur, on Bathing 104
Clark, Dr. on the climate of Italy 577
Clinical reports, Dr. Black's 357
Clutterbuck, Dr. on fever 64
Climate of France and Italy 577
Coindet, Dr. on hydrocephalus 505
Complaint, language of, in pain 158
Congestive typhus 417
Constipation of the bowels, fatal 242
Consumptive patients, Dr. Clark on 577
Convulsive ebriety 106
Cooke, Dr. on hydrocephalus 505
Cooper, Mr. Astley, on the urethra 453
——— on encysted tumours 457
——— on dislocations 557
Crampton, Dr. on dropsy 7
——— on the pancreas 263
——— on the mucous membranes 267

4 X

### INDEX.

D.

Davis, Dr on infantile diseases 151
Dendy, Mr. on infantile diseases 157
Desgaultiers, his idea of fever 589
Dickenson, Mr. on yellow fever 186
Diabetes mellitus, Dr. Black on 364
Dickson, Dr. on fever wards 104
——— on phlegmatia dolens 116
Digestive organs, morbid state of 371
Dilator, description of a new 274
Dislocations of the ancle 557
Diuretics in gout 378
Douglas, Dr. on the fœtus 100
———, Mr. on medical topography 293
Dropsy, Dr. Crampton on 7
Dysentery, Mr. Ballingal on 31
———, Mr. Bampfield on 303
———, Dr. Bateman on 351
Duncan, Dr. on fever 58
Dunglison, Mr. on moxa 497

E

Ear, severe inflammation of 236
Eczema rubrum 643
Elephantiasis 641
Emphysema of the lungs 476
Epidemic cholera of India 547
Epilepsy, Mr. Mansford on 216
Erysipelas, Dr. Weatherhead on 282
——— Mr. Wilson on 433
Extra-uterine conception 132

F

Fever, Mr. Ballingal on 27

—— in Great Britain 58

——, Dr. Clutterbuck on 64

——, Dr. Duncan on 58

——, Dr. Percival on 80

——, Dr. Hildenbrand on 83

——, Dr. Welsh on 278

——, Dr. Armstrong on 401

——, Dr. Jackson on 529

Fœtus, evolution of 100

Foster, Dr. on the heart 440

Fuzzel, Dr. ruptured uterus 260

G.

Gastrodynia 349

Gout, a preservative 340

——, Dr. Scudamore on 367

——, treatment of 377

——, chronic 384

——, retrocedent 388

Granville, Dr. on Prussic acid 269

Grattan, Dr. on inflammation 237

Gravel, Dr. Majendie on 287

———, Mr. Brande on 291

Griffith, Mr. on fever in Canada 295

Guthrie, Mr. on iritis 600

H.

Hæmoptysis caused by worms 241
Hall, Dr. on the mimoses 535
Hand, on diseases of the 444
Heart, curious disease of the 135
——, pathology of 489
Hemerailopia 602
Hieres, climate of 581
High operation for stone 393
Hildenbrand on fever 83
Howship, Mr. on bones 43
Hunterian society 158
Hydrencephalus, Dr. Yeats on 98
Hydrocele, Mr. Wood on 48
Hydrocephalus cured by operation 57
———, Drs. Cheyne, Vaidy, Coindet, Yeats, and Cook, on 505
Hydrophobia 450
Hydrops ovarii 451
Hydrophthalmia, cases of 142
——— 451
Hydrothorax, pathology of 481
Hypertrophia simplex 489
Hystero thecotomy, case of 132

I & J.

India, epidemic cholera of 547
Inflammation, Dr. Grattan on 237
Ingurgitation, on 106
Ileus, practical researches on 613
Innominata tied by Dr. Mott 147
Intestinal worms, Dr. Barry on 266
Intus-susception 619
Jackson, Dr. on fever 529
Jardine, Mr. letter from 496
Johnson, Dr. on the death of Dr. Blair 133

K.

Kennedy, Dr. case of shark-bite 51
——— on Hydropthalmia 142
Kirby, Mr. on surgery 105
——— on luxations 460

L.

Lachrymal organs, on the 101
Laennec on diseases of the chest 461
Larrey, Baron, on pain 158
Laryngitis, Dr. Hall on 607
Leeching in gout 380
Literary diseases 405
Lithotomy 631
Liver complaints, Mr. Ballingal on 41
Liver, suppuration of 447
Local blood-letting, on 114
Locher, Dr. Cæsarian operation 257
London, diseases of 333
Lotion, Dr. Scudamore's, in gout 382
Lungs, Laennec on the 461
Lunatics, horrid state of, at Turin 592

M.

Mackenzie, Mr. on the eye 101
——— Dr. on mineral waters 296
Majendie, Dr. on gravel 287
Mansford, Mr. on epilepsy 216
Mantell, Mr. on fracture of the pelvis 448
Marseilles, climate of 580

### INDEX.

Maunoir on the cautery 248
Medical topography of Canada 293
Midwifery, Mr. Power on 221
Mimoses, Dr. Hall on 535
Mineral waters 296
Morbid anatomy, cases of 136
Mott, Dr. ligature of the innominata 147
Mucous membranes, disease of 267

N.

Nævus, Mr. Wardrop on 49
Nice, climate of 581
Nicholl, Dr. on ol. terebinth. 261
———— on angina pectoris 262
Nitro-muriatic acid bath 350
Norman, Dr. on periodical diseases 138

O.

O'Brien, Dr. on diseases of the brain 235
Œdema of the lungs 483
Ophthalmia, Mr. Wardrop on 598
———— periodica 601
Ophthalmic surgery 324

P.

Pain, language of complaint in 158
Palmer, Dr. extirpated the parotid gland 254
Pancreas, disease of 263
Paracentesis, Dr. Archer on 250
Parkinson, Mr. on fever wards 103
Parmly, Mr. on the teeth 607
Parotid gland extirpated 253
Parry, Dr. on the circulation 201
Pathology of the heart 489
Pavia, school of medicine in 591
Pearkes on literary diseases 405
Pectriloquism 462
Pelvis, fracture of 448
Penis, Mr. Todd, on diseases of the 52
Percival, Dr. on fever 80
———— on the pancreas 263
Periodical diseases, remarks on 138
Peritonitis chronica 358
———— 629
Philip, Dr. his physiological discussion 657
Phlegmatia dolens 116
Pleurisy, Pathology of 479
Pneumonia, pathology of 474
Port, Dr. case of aneurism 49
Poultices in gout 381
Power, Mr. on midwifery 221
Prostate gland, enlargement of 574
Proximate cause of gout 373
Prussic acid, Dr. Granville on 269
Psoas abscess, Mr. Wilmot on 252
Pulmonary apoplexy 485
Pyloric ulceration 445

R.

Rennell, Mr. on scepticism 1
Ridley, Mr. on berri berri 435
Rheumatic ophthalmia 598
Rheumatism, acute 449
Robertson, Dr. on dysentery 319
Rumsey, Mr. on hæmoptysis 241

S.

Scatchard, Mr. on puerperal fever 556
Scepticism, Mr. Rennell on 1
Scott, Dr. on puerperal fever 555
Scudamore, Dr. on gout 367
Shark-bite, Dr. Kennedy's case of 51
Shoulder, dislocation of 460
Simpson, Mr. on hemerailopia 602
Souberbielle on lythotomy 393
Specifics in gout 378
Stethescope of Laennec 462
Stoker, Dr. on constipation 242
———— on apoplexia cephalica 244
Stone removed from the bladder by Mr. Arnott's dilator 277
————, high operation for 393
Stricture, Mr. Arnott on 273
Supplemental review 595

T.

Teeth, Mr. Bell on 604
Testicle, disease of the 575
Tetanus, Dr. Stewart on 646
Thackrah on the blood 296
Thorax, obscure disease of 611
Tic douloureux, remarkable case of 354
Todd, Mr. on the penis 52
———— on disease of the hand 444
Transactions of societies 231
Travers, Mr. on aneurism 349
Tropical dysentery, treatise on 303
Tubercles of the lungs 463
Tumours, Mr. Cooper on 457
Turpentine, oil of, Dr. Nicholl on 261
Typhus, Dr. Armstrong on 401
————, congestive 417

U.

Urea deficient in bilious urine 365
Urethra, stricture of 273
————, Mr. Cooper on 453
Urine, its specific gravity in gout 373
Uterus, ruptured, case of 260

V.

Vaidy, Dr. on hydrocephalus 505
Villa Franca, climate of 583
Volvulus 620
Vose, Dr. on Hydrencephalus 57

W.

Wadd, Mr. his cases in surgery 572
Wardrop, Mr. on Nævus 49
———— on Ophthalmia 598
Watts, Dr. on apoplexy 437
———— on the liver 447
Weatherhead, Dr. on erysipelas 282
Welsh, Dr. on blood-letting 278

Williams, Mr. on the heart 441
Willmott, Mr. on psoas abscess 252
Wilson, Mr. on the blood, &c. 432
Wood, Mr. on hydrocele 48

Y.

Yeats, Dr. on hydrencephalus 98—505
Yellow fever, Mr. Dickenson on 186

### *General List of Works analytically reviewed in the two first Volumes.*

VOL. I.

Armstrong, Dr. on Scarlet Fever 46
Percival, Dr. on Fever 63
Messrs. Cooper and Travers' Surgical Essays, Part I. vol. I. p. 79, 87—Vol. II. p. 460, 557.
Crampton, Dr. on Periostitis 95
Broussais, Dr. on Excitement, vol. I. 103
Dublin Hospital Reports and Transactions Vol. I. 190, 200, 212, 220, 541—Vol. II. p. 8, 52, 231, 242, 244, 253, 261, 263, 267, 435, 489, 444
Home, Sir E. on the Prostate Gland 206
Bell's, Mr. Charles, Quarterly Reports, 209
Newnham, Mr. on Inversio Uteri 222
Wilson, Dr. on Morbid Sympathies 341
Reid, Dr. on Tetanus, &c. 353
Balfour, Dr. on Emetic Tartar 358
Bateman, Dr. on Fever 360
Mansford, Mr. on Consumption 375
Philip, Dr. on the Vital Functions 394
Ayre, Dr. on Marasmus 399
Carmichael, Mr. on the Venereal 418
Orfila on Poisons 505
Medico Chirurgical Transactions 516
Bradley, Dr. on Stridor Abdominalis, 529
Hallaran, Dr. on Insanity 555
Brodie, Mr. on diseases of the Joints, 567
Armstrong, Dr. on Puerperal Fever, 583

VOL. II.

Rennell, Mr. on Scepticism 1
Crampton, Dr. on Dropsies 8
Ballingall, Mr. on Fever, &c. 2
Medico-Chirurgical Transactions 47
Duncan, Dr. on Fever 58
Perceval, Dr. on Fever 80
Hildenbrand on ditto 83
Adams, Sir W. on Cataract 86
Baron, Dr. on tuberculated Accretions 169
Dickenson, Mr. on Yellow Fever 186
Blane, Sir G. on ditto 194
Parry Dr. and Mr. Bell on the Circulation 201
Mansford, Mr. on Epilepsy 216
Power, Mr. on Midwifery 221
Transactions of Societies 231
Granville, Dr. on Prussic Acid 269
Arnott, Mr. on Stricture 273
Welch, Dr. on Blood-letting 278
Weatherhead, Dr. on Erysipelas 283
Magendie, Dr. on Gravel 287
Douglas, Mr. on Canada 293
Thackrah, Mr. on the Blood 296
Bampfield, Mr. on Dysentery 306
Bateman, Dr. on the Diseases of London 333
Black's, Dr. Clinical Reports 357
Scudamore, Dr. on the Gout 367
Carpue, Mr. on Lythotomy 393
Armstrong, Dr. on Typhus 401
Transactions of Societies 435
Works on Hydrocephalus 505
Jackson, Dr. on Fever 529
Hall, Dr. on the Mimoses 535
Epidemic Cholera of India 547
Cooper, Mr. A. on Dislocations 557
Clark, Dr. on Climate 577
Supplemental Review of Practical Medicine, for the first Quarter of 1820, 595

REFERENCE TO THE PLATE AND DRAWING.

Mr. Kirby's Luxation of the Shoulder-Joint - - - - - - 460
Mr. Wadd's Case of diseased Bladder - - - - - - - - 575

END OF VOL. II.

PRINTED BY W. THORNE, RED LION COURT, FLEET STREET.

### SUBSCRIBERS

TO THE

### Medico - Chirurgical Journal.

Adams, Sir W. Albemarle Street
Anderson, Dr. R. N.
Alcock, Mr. Piccadilly
Ayres, Mr. Mary-le-bone Street
Alexander, Mr. John, Surgeon, Newbury, Berkshire
Ange, Mr. James Surgeon, Fareham, Hants
Anderson, Mr. W. (2) Surgeon, H. M. S. Grasshopper
Addison, Mr. J. Surg. Burnham
Allen, Mr. J. Surg. Weymouth
Anderson, Mr. Surgeon, Bouverie Street
Andrews, Mr. Surg. Richmond
Allan, Mr. J. Surgeon, Jermyn-street, St. James's
Arnott, Dr. Bedford-square
Ashwell, S. Esq. Westminster Lying-in Hospital
Armstrong, Dr. J. Southampton-row
Burnett, Dr. Chichester
Bampfield, Mr. Bedford-street
Baynton, Mr. Bristol
Barry, Dr. Clifton
Burnaud, Mr. Chichester
Bishop, Mr. Newman-street
Bartlett, Mr. Ipswich
Bell, Mr. Newry
Bennet, Mr. Shifnal, Salop
Byass, Mr. Arundel
Barton, Mr. Manchester
Bampfield, Mr. 31st Regim.
Berryman, Mr. Penzance
Bowman, Mr. D. Harley-street
Brigham, Mr. Manchester
Balantine, Mr. Middlesex Hospital
Best, Mr. Tavistock-street, Covent Garden
Blacklock, Mr. Surg. Dumfries
Boylston Medical Society, United States
Bruce, N. Esq. Surgeon, Royal Military College, Sandhurst
Birckhead, Dr. Lennox, Baltimore, United States
Burchell, Mr. Mare-st. Hackney
Bushell, Mr. Great Russell-street, Covent Garden
Bailey, Dr. Reading, Berkshire
Barry and Co. Broad-st. Buildings
Bent, Mr. Diss
Bullen, Mr. Surgeon, Ipswich
Bullen, Mr. George, Surgeon
Burrows, Dr. Gower-street
Bath, Jacob, Esq. Surgeon to the Forces
Bean, Mr. Surgeon, Camberwell
Boulton, Thomas, Esq. Thornbury, near Bristol
Baron, Dr. John, Gloucester
Bartley, Dr. Assistant Surgeon to the Forces
Bath, Dr. Sen. King-street, Portman-square
Beatty, Wm, M. D. F. R. S. &c. Physician of the Fleet, &c. London
Belcombe, Dr. H. S. Newcastle-under-Line, Staffordshire

Blackett, Charles Powel, Park-street, Grosvenor-square
Blake, Mr. Chemist, Piccadilly
Broadfoot, Dr. Inspector General of Health, Corfu
Burnidge, Mr. T. D. Worcester
Buxton, J. Esq. Gatton Park, Ryegate
Bell, Mr. W. Assistant-Surgeon, Trincomalee Hospital, Ceylon
Bell, Mr. Alex. Member of the Royal College of Surgeons, London, Surgeon to the Dundee Royal Infirmary
Boutflower, C. Esq. Surgeon to the Forces, Colchester
Bree, Dr. Robert, George-street, Hanover-square
Breschet, M. M. Chef des Travaux Anatomiques, Hospice de Perfectionment a Paris
Buchanan, Mr. Surgeon, Terrace, City-road
Byass, Mr. Surg. Crookfield
Bowen, Dr. J. Caermarthen
Bradley, O. Esq. Surgeon, R. Westmoreland Milit. Appleby
Bisset, Charles Edmund, Surgeon, Peckham
Broughton, S. Esq. Falmouth
Braine, J. W. Esq. Oxford
Bartlett, P. Esq. Nottingham-place
Callow, Mr. 20th light dragoons
Cooper, Mr. Portsea
Cuthbert, Mr. Suffolk
Candler, Mr. Grundesburgh
Crawford, Mr. Southampton
Cunningham, Mr. Plymouth
Carbutt, Dr. Manchester
Clifton, Mr. London
Curtis, J. H. Esq. Soho-square
Calley, Mr. Surgeon, East Essex Militia, Colchester
Carmichael, R. Esq. Dublin
Clarke, Sir Arthur, Dublin
Clarke, Mr. Windmill-street Haymarket
Campbell, Dr. Dougal, Picardy-place, Edinburgh
Clarke, J. F. Esq. Surg. Currie
Corsan, John, Esq. Royal Navy
Cosgrave, Mr. Batavia
Clements, Mr. Wadebridge, Cornwall
Coyne Phineas, Esq. Welbeck-st.
Crane, William, M. D. Senior Physician to the Boston General Dispensary
Crucifix, Mr. Surgeon, Bouverie-street, Fleet-street, London
Cary, Mr. J. W. Surgeon, Trowbridge
Chatham Medical Society
Clark, Dr. James, Resident Physician, Rome
Copland, Dr. 2, Walworth Terrace
Curry, Dr. (since dead)
Chapman, Mr. Surgeon, Windsor
Clarke, Dr. J. Cuthbert, Smyrna
Coley, J. M. Esq. Bridgnorth
Colquhoun, Dr. Craven-st. Strand
Cornish, Mr. James, Falmouth
Crichton, Dr. West Indies
Chambers, Mr. Charles, Member of the Royal College of Surgeons London, Leamington-spa
Creasy, Mr. Surg. Eden-bridge, Surrey
Cooper, Astley, Esq. Spring-gardens
Chilvers, Samuel, Esq. New Burlington-street
Child, Mr. Assistant-Surgeon, Madras Establishment
Cooke, Dr. John, F. R. College of Physicians, Gower-street
Camm, W. Esq. Nottingham
Clarke, T. Esq. Newman's-row, Lincoln's-inn-fields
Crawford, Dr. Bath

Dickson, Dr. Clifton
Denmark, Dr.
Dewar, Dr. Edinburgh
Davie, Mr. Ipswich
Davidson, Mr. Edinburgh
Davidson, Mr. Alexander
Davis, Dr. George-street

Daniels, Mr. Princes-street
Drew, Mr. James, Gower-street
Davies, Dr.
Dobson, Dr. Chatham Division of Marines
Dillon, Mr. Surgeon, Lambeth-terrace
Davidson, Dr. Nottinghamshire
Davies, Mr. W. Orchard-st. Portman-square
Denecke, Dr. Isle of Wight
Downe, Dr. J. S. Resident Physician, Florence
Ducamp, M. Docteur en Medecine, Rue St. Martin, à Paris
Dugdale, Mr. Blackburn, Lancashire
Dawson, Ensign, St. Kitts, West-Indies
Delph, Mr. Surgeon, Northumberland-place, Edmonton
Dobie, Mr. Exmouth-street, Spa-fields
Draper, Mr. Surgeon, Burford, Oxfordshire
Dwyer, Dr. John, Physician to the Forces, Ceylon
Davies, Mr. W. S. G. Surgeon, Poland-street
Davidson, Dr. J. M. Hanley, Staffordshire
Darrack, Mr. Philadelphia
Davies, Mr. Conduit-street
Dickenson, Mr Milcham
Dickenson, Mr. Sloane-street

Ellerby, Mr. Cannon-street
Evans, Dr.
Elliot, J. Esq. Surg. to the Forces
Emerson, Dr. A. L. Brompton

Ford, Mr. Southsea
Felix, Dr. Bristol
Fletcher, Mr. Arundel
Fairchild, Mr. Rochford, Essex
Fallofield, Mr. Albemarle-street
Fiddes, Mr. 85th Regiment
Forbes, Dr. Penzance
Fraser, Rev. Simon, Edinburgh
Freer, John, Esq. Chelsea
Freshfield, Mr. P. W. Surgeon, Oakley, Essex
Fox, Mr. Surgeon, Huntingdon
Filson, Mr. Robert, Honourable East India Company's Service, Madras
Farnden, Joseph, Esq. Surgeon, 70th Regiment, Canada
Foster, Dr. Wm. Member of the Royal Medical Society, Edinburgh, Oakes, near Sheffield
Fitzpatrick, Mr. Royal Artillery, Woolwich
Fewster, Mr. Thomas, Surgeon, Thornbury, Gloucestershire
Forbes, Dr. Argyle-street
Forman, Mr. G. Ellery, Member of the Royal College of Surgeons, London
Fothergill, Dr. W. late Editor of the Medical and Physical Journal, Jamaica
Fosbrooke, Mr. London
Foot, Mr. Jessé, Jamaica
Foot, Mr. George Forrester, Jamaica
Fearn, Mr. T. Huntsvile, State of Alabama, U. S.
Franklin, H. Esq. Surgeon of the 37th Regt. Montreal
Forbes, Dr. Chatham

Grant, Dr. Edinburgh
Grebke, Mr. R. N.
Gunning, Deputy-Inspector
Gardener, Rev. F. Neston
Gray, Dr. R. N. Haslar
Grattan, Dr. Dublin
Gunning, Mr. John, Surgeon to Saint George's Hospital
Grabham, Mr. J. Surg. Rochefort
Gordon, Dr. Alexander, Finsbury-square, London
Graham, Dr. W. late of Jamaica
Gilbert, Dr. Theodore, Bermuda
Goolden, R. Esq. Maidenhead
Guthrie, J. G. Esq. Berkley-st. London
Gronous, Mr. West Indies
Grant, Mr. Surgeon, Bristol

Green, Mr. W. Surg. Durham
Green, Dr. G. Yarm, Yorkshire
Green, Mr. Josephus Septimus, Surgeon, Durham
Grey, Sir Thomas, M. D.
Guy, Mr. John Member of the Royal College of Surgeons, London
Gilbert, Mr. James, Surgeon, Brompton, Rochester
Gorman, Mr. Surgeon, Castle-st.
Gordon, Dr. Blenheim

Hall, Mr. R. N.
Howell, Dr. Clifton
Harding, Mr. Kentish-town
Hunt, Mr. Bombay
Highley, Mr. Elland
Hennen, Inspector, Edinburgh
Hogg, Mr. Littlehampton
Hall, Dr. John, Berwick
Hutchison, Mr. A. C. London
Harper, Mr. W. Manchester
Hendrickson, Mr.
Howel, Dr. Neath
Howship, Mr. George-street
Hastings Dr. Charles, Physician to the Worcester Infirmary
Higgins, Mr. F. Little Russell-street, Covent-garden
Haden, Mr. Sloane-street
Halkyard, Edward, Esq. Oldham, Lancashire
Hamilton, Dr. Finsbury-square, London
Hillyar, Dr. Royal Navy
Hughes, Dr. Physician to the Forces, Barbadoes
Hatfield, Mr. Thomas, Herts
Higgs, Mr. John Maidenhead, Berks
Hingeston, Mr. J. Surgeon-Apothecary, Coleman-st. London
Hollis, Mr. W. M. Surgeon
Holt, Mr. Surgeon, Tottenham
Hands, Mr. B. Turnham-green
Harshaw, Mr. Surgeon, R. N.
Hillier, Mr. H. Dean-st. Borough
Hora, Mr. James, Surg. Harwich
Hull, Dr. Manchester
Hamet, Dr. East Indies, with the Commander in Chief
Harold Philip, Esq. Surg. Holt, Norfolk
Hassel, Dr. and Surg. Bolougne Sur-Mer
Hume, Dr.
Hunter, Dr. Richmond
Howard, Dr. William, Baltimore, United States
Hall, Mr. Robert, Durham

Illingworth, Mr. William, Surgeon, Hackney
Johnston, Mr. Dowgate-hill
Jubb, Mr. Halifax
Johnson, Edw. M. D. Weymouth
Johnson, D. Esq. Torrington, Devon
Jones, Mr. Surg. Princes-street, Cavendish-square
Jordan, Mr. St. James's-square, Manchester
Jenkins, Mr. Fossy, Surg. Sutton Valence, near Maidstone, Kent
Johnston, Dr. Ireland
Jones, Dr. George Haines, Portsmouth
Jouenne, Dr. Rue de L'Orangerie, Brussels
Jackson, Dr. Jamaica
Jardine, W, Esq. Surgeon, R. N. Dumfries
Jenner, Dr. Edward, Berkeley, Gloucestershire
Johnston, Dr. Bengal Medical Establishment

Keddell, Mr. Portsea
Keenbyside, Mr. R. H.
Kedgell, Mr. Richard, Surgeon, Wellington, Somerset
Keith, Dr. James, Trinidad
Kirby, J. Esq. Dublin
Kerr, Robert, Esq. Portobello, South America
Kennedy, Mr. C. (St. Thomas's Hospital) Alverstone, Lancashire

Lempriere, Dr. Isle of Wight
Lynn, Mr. Woodbridge
Little, Dr. Tandragee
Lane, Mr. Arundel
Leslie, Mr. R. N.

Lyne, Mr. Emsworth
Larver, Mr. 18th Regiment
Lillies, Mr. George, R. N.
Luke, Dr. Argyle-street
Lyon, Dr. Manchester
Locock, Mr. Italy
Lodge, Mr. Haymarket
Lyster, Mr. 7th Dragoon Guards
Lanyon, Mr. R. Lostwithiel
Lithgow, Mr. Andrew, Surgeon, Weymouth
Lucas, Dr. C. E. Hatfield
Lewis, G. Esq. Surg. Sudbury
Lynn Regis Medical Book Society
Leggett, Mr. Wm. St. Thomas's Hospital
Litchfield, Mr. Twickenham
Lutener, Mr. Bridge-north

M'Donnel, Dr. Belfast
Medical Society, Chichester
——————, Halifax
Moulson, Dr. Halifax
Morrison, Dr. Newry
Muttlebury, Dr. Bath
Meek, Mr. Edinburgh
Medical Book Club, Edinburgh
Milne, Dr. Edinburgh
M'Carrogher, Dr. R. N.
Mitchell, Mr. Keighley
M'Arthur, Dr. Deal
Medical Society, Deal and Sandwich
——————, Portsmouth
Medical Book Society, Kingston, Jamaica
Moffat, Mr. Panton-square
Mardel, Mr.
M'Grigor, Sir James, M. D.
Manchester Infirmary Committee
Medico-Chirurgical Societ. Lond.
Mortimer, Mr. Torrington
M'Leod, Mr. John, R. N.
Merriman, Dr. Half Moon-street, Piccadilly
Mould, John, Esq. Surgeon, Oundle, Northamptonshire
May, S. Esq Surgeon, Ilfracombe
Metcalfe, Thomas Thompson, Sheffield
Montgomery, Mr. A. Surgeon, Paris
Moore, Mr. Surgeon, Woodbridge
Mullins Mr. John
Murray, Mr. John, Edinburgh
M'Ternan, P. Surgeon, R. N.
Marshall, Mr. Assist. Surg. R.N.
Maysmer, Mr. R. Surgeon, Ashborne, Derbyshire
Medical Society, Bolt-court
Mitchel, Mr. Surgeon, R. N.
Monteath, Dr. G. C.
M'Whirter, Dr. F. Newcastle-upon Tyne
M'Dougle, Dr. Paris
Magrath, George, M. D. F.R S. and L.S. Physician to the Dock and Stonehouse Dispensary, Plymouth
Mantell, Gideon, Esq. F. L. S. Surgeon to the Royal Ordnance Hospital at Ringmer, Castle-place, Lewes
Marshall, Mr. Surgeon, Belfast
Macewan, Mr. George, Surgeon Grenada
Morel, Mr. New Bond-street
Moon, Mr. Medical Staff
Moffatt, Mr. Surgeon, Long-lane
Money, Mr. Hanover-street, Hanover-square
M'Leod, Dr. Ophthalmic Hospit.
M'Knight, Dr. W.G. Jamaica
Mathews, Mr. Thomas Leman, H. C. S. Prince Regent
Morris, Mr.
M'Millen, Mr. Surgeon, R. N.
Morrison, Mr. W. Surgeon, H.P. 12th Foot, Bristol
Millington, Mr. Titchfield-street

Nagle, Dr. R. N.
Nowood, Mr. Dover
Nicol, Dr. Sierra Leone
Norman, Mr. Langport
North, Mr. Seymour-place
Newenham, Mr. W. Farnham
Newsom, Mr. Surg. Rochester
Nunn, Mr. J. Surg. Holbrook
Nussey, Mr. St. James's-street
Neilson, Dr. Trinidad

Newry Medical Society

Osborne, Mr. R. N.
Outhwaite, Mr. Bradford
O'Meara, Mr.
Ogle, Richard, Esq. Great Russell-street, Bloomsbury
O'Malley, J. Esq. Surgeon of the 11th Light Dragoons Bengal
Osborne, Mr. Surgeon, Gracechurch-street

Philip, A.P.W. M.D. Worcester
Plowden, Dr. Midhurst, Sussex
Porter, Mr. Portsea
Percival, Dr. G. Bath
Porter, Dr. Bristol
Pineo, Mr. R. N.
Pink, Mr. Portsea
Pesket, Mr. East Aspling
Palmer, Dr. Tamworth
Punshon, Mr. Ceylon
Paxton, Sir William, Piccadilly
Pool, John, Esq. Walton-upon-Thames
Powell, Mr. Newman-street
Parkinson, Mr. George, Racquet-court, Fleet-street
Perkins, Mr. Measham
Prevost, Dr. Switzerland
Price, Dr. W. United States
Price, Mr. W. Surgeon, R. N.
Pyne, Mr. William Collard, Surgeon, Wellington, Somerset
Parmly, Mr. Surgeon Dentist, Pall Mall
Phillips, Mr. Samuel, H. M. S. Nimrod, Leith
Pierce, Dr. Edward, Wexford
Park, Mr. Surgeon, Kingsland-Road
Penrith Medical Society, Cornwall
Porter, Dr. Arthur, Little Hanover, New Hampshire
Powell, W. H. Esq. Surgeon, Manchester-st. Manchester-sq.

Quarrier, Dr. R. M. A
Quebec Medical Society

Ross, Mr. Stirling
Rogers, Mr. H. M. S. Minden
Robertson, Dr. Northampton
Rankin, Mr. Marchmont-street
Robertson, Dr. Edinburgh
Risk, Dr. Saltash
Roberts, Mr. Manchester.
Roscoe, Mr. Richard
Royle, Mr. Sloane-street
Rudge, Mr. Middlesex Hospital
Runciman, John, R. N.
Rodmel, J. Esq. Surgeon, R. N.
Russel, Mr. G. I. London
Ryland, Mr.
Royal Ordnance Medical Society, Woolwich
Russell, Mr. Surgeon, Mount-st.
Reilly, J. Esq. Plymstock, near Plymouth
Ring, J. Esq. New-street, Hanover-square
Robinson. Mr. Holles-street, Cavendish-square
Robinson, Mr. Mark, Beverly, Yorkshire
Roberton, J. Esq. Hospital Assistant to the Forces
Russell, Mr. R.
Reay, Mr. Surgeon
Rodd, Mr. Surgeon, Hampstead
Rouzet, Dr. Rue des St. Peres, à Paris, in Exchange for "La Revue Medicale, &c."
Rudland, W. H. Esq, Surgeon, Royal Navy, Dartmouth
Rutherford, Dr.
Royal Institution, Albemarle-st.
Riley, Edward, Esq. Sydney, New South Wales
Rodgers, Mr. J. Settle, Yorkshire

Smith, Mr. Malta
Sharpe, Mr. Bushire, Persia
Stewart, Mr. H. M. S. Bulwark
Sims, Mr. Southsea
Sutcliffe, Mr. Rochedale
Sanden, Dr. Chichester
Stebbing, Mr. Ipswich
Scudamore, Dr. Wimpole-street
Sanders, Dr. Edinburgh

Scott, Mr. Edinburgh
Steel, Dr. Walter, Edinburgh
Stewart, Mr. Edinburgh
Shaw, Mr. Halifax
Seeds, Dr. Stafford, America
Stewart, Mr. New Forest
Skair, Mr. Castle-street, Ed.
Stewart, Mr. H. M. S. Carron
Shepherd, Mr. Witney
Shoveller, Mr. R. N.
Simpson, Mr. James, Edinburgh
Sankey, Mr. Dover
Stilon, Mr. Joseph, Malta
Smalley, Mr. Robert, Richmond Buildings
Sprague, J. H. Esq. Surgeon, North Curry, Somerset
Stedman, James, Esq. Guilford
Stokoe, Dr. R. N.
Sainsbury, W. M. D. Corsham, Wilts
Sherwood, Ralph, Esq.
Shooldred, Mr. John G. Charles Town, South Carolina
Stacy, Mr. London Hospital
Stewart, Mr. St. James's-square, Manchester.
Swift, J. S. Esq. Surgeon, R. N.
Shaw, Mr. Surgeon, Anatomical Theatre, Great Windmill-st.
Shute, Mr. Surgeon, Gosport
Sproule, Dr. Bombay
Stowe, Mr. W. Surgeon, Buckingham
Scalt, Mr. Surgeon, Easingwold, Yorkshire
Scott, Dr. Russell-square
Shearman, Dr. W. Northampton-square
Slow, D. Esq. Royal Horse Gds.
Smart, Mr. Frith-street, Soho
Stenson, Dr. Burton-on-Water, 2 Copies
Stolterforth, S. Esq. Bolton-st. Piccadilly
Sawyer, Mr. Margate
Scatchard, Mr. John, Surgeon, East Keswick, near Leeds
Scott, Dr. John
Shipton, Mr. Chipping
Stevenson, Mr. M.R.C.S. Lond.
Stanton, Dr. R. N. Moulton, Northamptonshire
Steart, Augustus W. Bath
Squib, Mr. Saville-row

Tickell, Mr. E. Burton, Somerset
Todd, J. T. Esq. Havre-de-grace
Thompson, Dr. Belfast
Travers, Mr. Bagholt
Thomas, Mr. H. L. Leicester-sq.
Tedlie, Mr. Beresford, 85th Reg.
Tedlie, Dr. Cape Coast Castle, Africa
Twemlow, Mr. Surg. Northwich
Tobin, Mr. John, Surgeon
Thompson, Dr. Paris
Thompson, Dr. Conduit-street
Thompson, Mr. Merchant, West Indies
Travis and Dunn, Messrs. Scarborough
Trevosso, Mr. Surg. Falmouth
Taylor, Dr. David, Wotton-under-edge, Glostershire
Thornton, Dr. Edward, Stroud
Turner, Mr. New-st. Baker-st.

Uwins, David, M. D. in Exchange for Medical Repository
Valentine, Mr. J. W. Surgeon, Sutton-in-Ashfield, near ansfield
Valentine, Mr. John, London
Veitch, Dr. Grafton-st. London
Vaile, W. Phelps, Esq. Member of the Royal College of Surgeons, Lond. Headcorn, Kent

Weir, Dr. Victualling Board
Williams, Dr. W. H. Ipswich
Wardrope, Mr. Arundle
Williams, Mr. G. Portsea
Webster, Mr. 51st. Regt. L. I.
Warden, Mr. R. N.
Willes, Mr. Ratcliffe Highway
Walker, Dr. John, Walbrook
Wake, Dr. Bartholomew, Somerset
Wallis, Mr. Charles, Lincoln's-Inn

White, Mr. B. at Mr. Brookes's
Whitney, Mr. 85th Regiment
Whitter, Dr. Fellow of the Royal College of Physicians, Bridge Street, Blackfriars
Wilmore, Mr. John, Surgeon, Worcester
Worcester Medical and Surgical Society, 2 Copies
Wright, Mr. George Street, Portman Square
Warwick, Mr. Richard, Assistant Surgeon, R. N.
Wight, Dr. Robert, Madras
Willis, Mr. Ratcliffe Highway
Wight, Mr. Chertsey
Williams, Mr. William, Llandovery, South Wales
Walton, Mr. J. E. Surg. Guilford-street
Wilson, J. Esq. F. R. S. George-street, Hanover-square
Wilson, Mr. Surgeon, Belfast
Wilmore, Edmund Wm. Member of the Royal College of Surgeons in London, Worcester
Williamson, Dr. Whitehaven
Wadd, William, Esq. Park-place, St. James's
Weatherhead, Dr. Montague-st. Montague-square
Williamson, Mr. Whitehaven
Whiteing, the Rev. Mr. G. B. Hitching, Herts
Walton, George, Esq. Surgeon, East India Company's Service
Wardrop, James, Esq. F. R. S. E. Charles-street, St. James's-sq.
Walker, Dr. Sanderson, St. Michael's, Azores
Warwick, Mr. R. N. Newfoundland
Whimper, Mr. G. M. Surgeon, Tillingham, Essex
Walton, Mr.
Webster, Mr. J. Leicester

Yates, Dr. T. Brighton
Young, Mr. W. Surg. Ballymena
York Hospital Library
Young, Dr. James Forbes, Paradise-place, Lambeth

The list of *additional* Subscribers will appear, as usual, every Quarter ; and the whole will be incorporated into a general list, Annually, prefixed to each Volume, with such alterations of titles, residence, &c. as the year may produce. It is to be observed, that what is meant by Subscribers, is merely those who take in the Journal, through the medium of their own book-seller, the same as any other periodical publication.

Additional Subscribers for next Quarter must send their names before the 15th of May, as the next number will be published on the 1st of June.

*N. B. Although each name comes in under its proper letter, yet the Arrangement is not strictly alphabetical in each letter. This will be rectified in the next general list.*

